# Diagnosis of Advanced Gastric Cancer Occasioned by Persistent Postpartum Fever: A Case Report

**DOI:** 10.7759/cureus.59563

**Published:** 2024-05-03

**Authors:** Takanori Sato, Hanae Kawamura, Gen Haba, Rie Oyama, Tsukasa Baba

**Affiliations:** 1 Obstetrics and Gynecology, Iwate Medical University, Yahaba, JPN

**Keywords:** gastrointestinal endoscopy, abdominal ultrasound, hyperemesis gravidarum, pregnancy-associated gastric cancer, gastric cancer, postpartum, persistent fever

## Abstract

Pregnancy-associated gastric cancer is extremely rare. In many cases, the cancer is already advanced at the time of diagnosis, and the prognosis is often poor. A 39-year-old primigravida, with dichorionic diamniotic twins, was admitted to our hospital for threatened preterm labor at 31 weeks of gestation. At 32 weeks of gestation, she developed a fever and tested positive for influenza A. She recovered from influenza A on the following day but had an emergency cesarean section for premature rupture of the membranes at 32 weeks of gestation. She was discharged on postpartum day six. Thereafter, she was again infected with influenza A. On day 18, she underwent an abdominal ultrasound revealing multiple mass lesions in the liver, because she had an uncomfortable upper gastric with persistent fever. She was referred to the internal medicine team, who made a diagnosis of stage IV gastric cancer. Importantly, non-specific symptoms and physical signs that are not explicable by the normal course of pregnancy may be recognized through conscientious history-taking and physical observations. If gastrointestinal symptoms are prolonged, or if symptoms such as severe weight loss, melena, a tender abdominal mass, or persistent and unexplained fever develop, an endoscopic assessment should be conducted to suspect other diseases. In addition, proactive ultrasound scanning, including the upper abdomen, may detect pregnancy-associated gastric cancer and lead to further in-depth investigations.

## Introduction

Gastric cancer was the fourth most common type of cancer among Japanese women in 2019. Its morbidity rate was higher in those aged ≥ 50 years, with its incidence in women of reproductive age being lower [1]. Pregnancy-associated gastric cancer, defined as a diagnosis of gastric cancer during pregnancy or up to one year after delivery, is extremely rare [2]. In most cases, the cancer is already more advanced at diagnosis compared with other women of the same age group, meaning that the prognosis is usually poor [3]. In pregnant women, hyperemesis gravidarum and gastrointestinal symptoms due to the enlargement of the uterus often delay the diagnosis of gastric cancer. In addition, postpartum women have fewer occasions to be examined in medical institutions, making the diagnosis of pregnancy-related gastric cancer even more difficult.

This report is a case of a patient with persistent postpartum fever who was detected with multiple liver tumors on abdominal ultrasonography by an obstetrician and diagnosed with advanced gastric cancer after consulting internal medicine.

## Case presentation

A 39-year-old primigravida with a medical history of polycystic ovarian syndrome and autonomic ataxia was admitted to our hospital for threatened preterm labor at 31 weeks of gestation. Family medical history included hepatitis B, prostate cancer, and hypertension in her father, and thyroid cancer in her mother.

The patient became pregnant after induction of ovulation by clomiphene citrate and human menopausal gonadotrophin at a local clinic and was diagnosed as carrying dichorionic diamniotic twins. She was also transferred to another hospital with a diagnosis of ovarian hyperstimulation syndrome, which improved with treatment. She received routine pregnancy checkups at that hospital. Symptoms of hyperemesis were evident at 15 weeks of gestation, and symptoms of gastric compression and vomiting were evident at 26 and 31 weeks of gestation. These symptoms did not worsen. At 31 weeks and five days of gestation, the patient was transferred to our hospital due to threatened preterm labor with dichorionic diamniotic twins. At 32 weeks and one day of gestation, the patient developed a fever and tested positive for influenza A. She was treated with oseltamivir, after which the fever resolved the following day. At 32 weeks and five days of gestation, an emergency cesarean section was performed due to premature rupture of the membranes. The patient did not have any trouble postoperatively and was discharged on postpartum day six.

She again developed a fever on postpartum day seven and experienced pain in her back and at the side of the abdomen, she presented at our hospital on postpartum day 10. Physical findings showed blood pressure was 121/81 mmHg, heart rate was 78 bpm, and temperature was 36.6ºC. The fever repeatedly resolved during the day and increased to over 38ºC at night, and upper respiratory symptoms of cough and nasal mucus continued. On physical examination, the abdomen was flat, soft, and not tender. Transvaginal ultrasound showed ascites, but no signs of uterine infection and normal findings in the uterine suture area. Suspecting pyelonephritis, an abdominal ultrasound was performed. Although there was no bilateral pyelectasis, a small mass was observed in the right lobe of the liver. There was no percussion tenderness of the liver. Blood tests indicated a mildly elevated white blood cell count (WBC) and C-reactive protein (CRP). As the patient again tested positive for influenza A, her fever was attributed to re-infection with influenza, and zanamivir inhalation therapy was started.

Because her fever persisted with temperatures of 37-38°C even after the end of zanamivir inhalation therapy, she was examined again in our department on postpartum day 18. Table 1 shows the results of the blood test from discharge to postpartum day 18.

**Table 1:** Results of blood test from discharge to postpartum day 18 and results of additional tumor markers tested on postpartum day 18. CEA: carcinoembryonic antigen; CA19-9: carbohydrate antigen 19-9; AFP: α-fetoprotein; PIVKA-II: protein induced by vitamin K absence or antagonist-II; sIL-2R: soluble interleukin-2 receptor.

Items	Results			Reference value
	Discharge	Postpartum day 10	Postpartum day 18	
White blood cell count	9180	9320	8350	3300-8600/μL
Red blood cell count	330	337	351	387-492 ×104/μL
Hemoglobin	10.6	10.7	10.7	11.6-14.8 g/dL
Platelet count	435	481	565	158-348 ×103/μL
Albumin	1.9	2.3	2.5	4.1-5.1 g/dL
Aspartate aminotransferase	24	46	34	13-30 U/L
Alanine aminotransferase	14	18	18	7-23 U/L
Lactate dehydrogenase	395	442	473	124-222 U/L
γ-glutamyl transpeptidase	24	29	71	9-32 U/L
Total bilirubin	0.3	0.3	0.4	0.4-1.5 mg/dl
Blood urea nitrogen	20.9	8.9	6.9	8-20 mg/dL
Creatinine	1.28	0.93	0.77	0.46-0.79 mg/dL
Serum Na	142	140	142	138-145 mmol/L
Serum K	4.2	3.7	3.8	3.6-4.8 mmol/L
C-reactive protein	3.11	5.34	6.28	0-0.14 mg/dL
			Additional testing	
CEA			11.1	0-3.4 ng/mL
CA19-9			464.0	≤37 U/mL
AFP			5.5	0-7 ng/mL
PIVKA-II			24	0-40 mAU/mL
sIL-2R			593	121-613 U/mL

**Figure 1:**
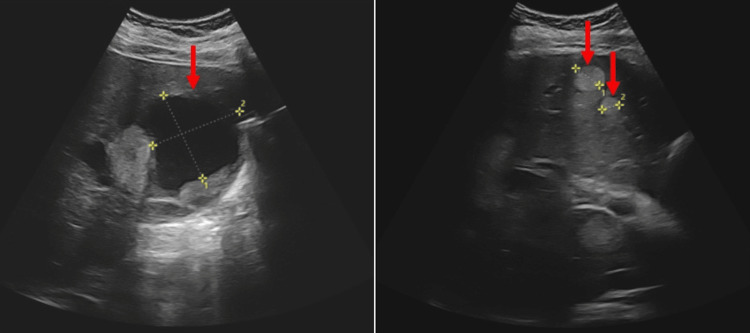
Abdominal ultrasound showed several cystic lesions and multiple mass lesions in the liver (red arrows).

In further investigations to identify the cause, transvaginal ultrasound showed that ascites was still present, and abdominal examination revealed mild tenderness and a palpable mass in the epigastric region. A repeat abdominal ultrasound examination revealed several cystic lesions with multiple solid portions throughout the liver (Figure 1).

**Figure 2:**
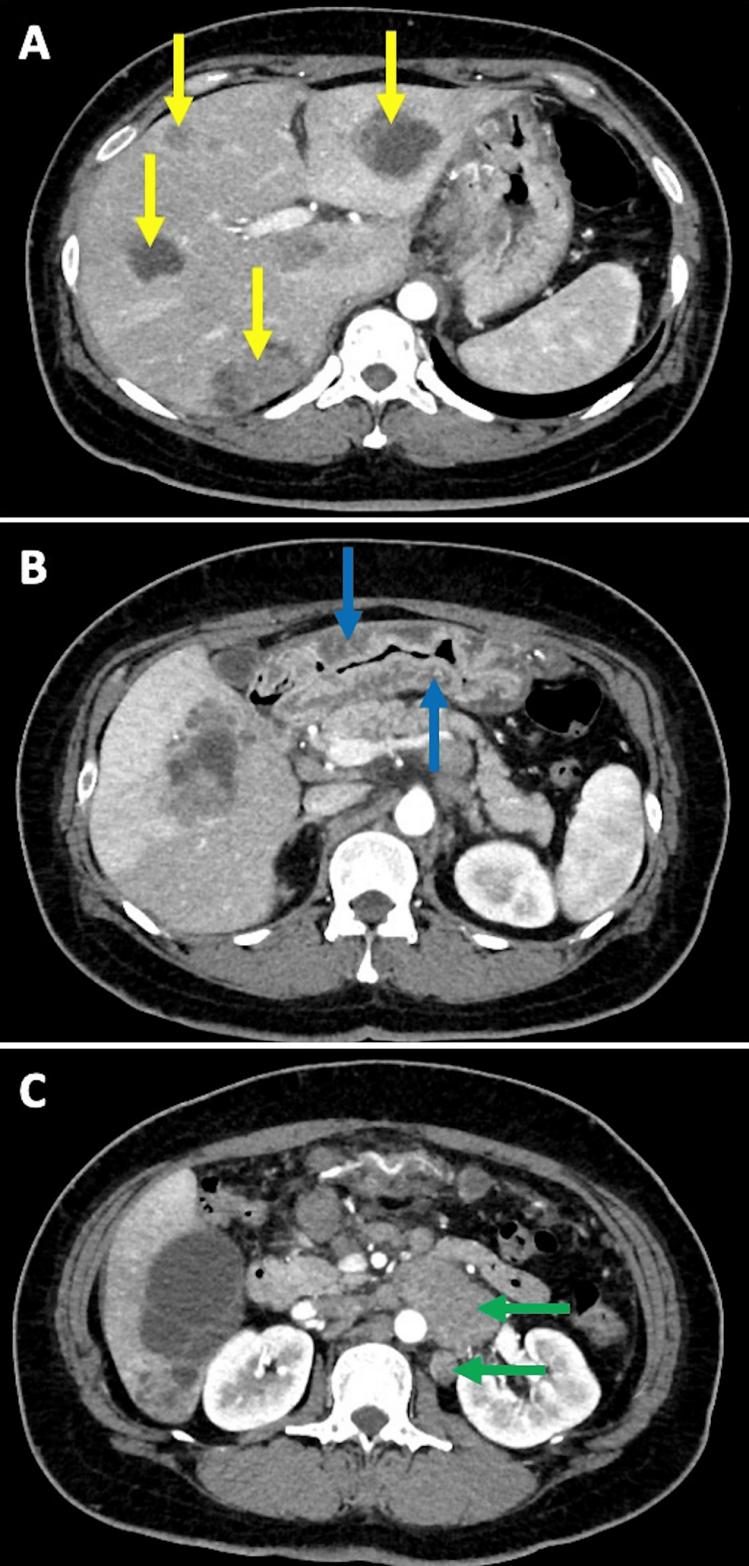
Contrast-enhanced computed tomography showed (A) multiple tumors with mixed cystic and solid components in the liver (yellow arrows), (B) gastric wall thickening (blue arrows), and (C) multiple enlarged lymph nodes (green arrows).

Contrast-enhanced CT was performed on the same day. Multiple tumors with mixed cystic and solid components were identified in both lobes of the liver, and multiple enlarged lymph nodes were observed in the supraclavicular, the hilum of the lung, around the abdominal aorta, and the hilum of the spleen, as well as splenomegaly, ascites, and gastric wall thickening (Figure 2).

**Figure 3:**
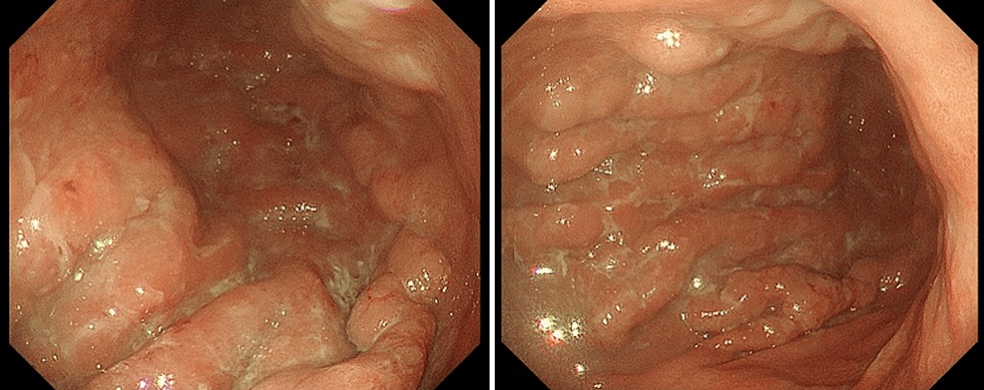
Upper gastrointestinal endoscopy revealed thickening of the gastric folds and poor extensibility of the gastric wall.

From the symptoms and imaging investigations, the patient was referred to specialists in both malignant lymphoma and multiple metastases of gastric cancer for further investigation. The soluble interleukin-2 receptor (sIL-2R) level was normal but carcinoembryonic antigen (CEA) and carbohydrate antigen 19-9 (CA19-9) were both elevated, and upper gastrointestinal endoscopy performed on postpartum day 20 revealed poor extensibility of the gastric wall and an elevated lesion at the same location (Figure 3).

Therefore, a diagnosis of Borrmann type 4 (diffusely invasive) gastric cancer was made. Histopathological analysis of the biopsy tissue showed adenocarcinoma and a diagnosis of stage IV gastric cancer was made.

Chemotherapy was started from postpartum day 29; S-1 plus cisplatin (SP), S-1 plus oxaliplatin (SOX), ramucirumab plus nab-paclitaxel (RAM+nab-PTX), nivolumab plus the investigational drug ONO-4578, trifluridine, and tipiracil hydrochloride were administered, but the disease progressed. Unfortunately, she passed away approximately 10 months after diagnosis.

## Discussion

The present case is an extremely rare case in which a patient was diagnosed with gastric cancer after the conscientious performance of abdominal examination and abdominal ultrasound because she exhibited the non-specific symptom of persistent postpartum fever. General abdominal pain is more likely recognized as a gastrointestinal disease. On the other hand, as the chief complaint of this patient was persistent postpartum fever, it was difficult to suspect gastric cancer. In previous reports, gastric cancer was diagnosed after delivery based on findings of nausea, vomiting, and abdominal pain, which were suspicious of gastrointestinal diseases [4], but the present case differs from previous reports in that the chief complaint was persistent fever. It is important to consider gastric cancer as a differential disease for fever even when gastrointestinal symptoms are unclear.

Pregnancy-associated gastric cancer is extremely rare, diagnosed in only 0.026%-0.1% of all pregnancies [2]. In a report by Song et al., 20 of 583 women diagnosed with cancer during pregnancy had gastric cancer [4]. In Japan, 12 deaths of pregnant or parturient women were related to malignant tumors between 2010 and 2016, of which four were due to gastric cancer, three were due to leukemia, two were due to ureteral cancer, and one each due to malignant lymphoma, brain cancer, and cervical cancer [5].

Although some cases of gastric cancer diagnosed during pregnancy may be detected by melena, jaundice, persistent upper abdominal symptoms, epigastric pain, hematemesis, or severe weight loss, most patients exhibit nonspecific symptoms such as abdominal pain, nausea and vomiting, and indigestion [2,4,6-8]. Symptoms of gastric cancer in non-pregnant women also include decreased appetite, stomach discomfort, nausea, and vomiting, and there is no significant difference in symptoms between pregnancy and non-pregnancy. Symptoms of pregnancy-associated gastric cancer resemble the hyperemesis gravidarum symptoms seen in the first trimester, and from the second trimester onward, they also resemble symptoms associated with the enlargement of the uterus, making gastric cancer during pregnancy extremely difficult to distinguish, and its diagnosis is often delayed. Furthermore, pregnancy-associated gastric cancer is characterized by hesitation to conduct examinations that are invasive or involve radiation exposure such as endoscopy and contrast-enhanced CT during pregnancy when only a few symptoms are present. This case exhibited symptoms of hyperemesis gravidarum at 15 weeks of gestation and of gastric compression and nausea at 26 weeks of gestation, but her symptoms were considered to fall under the category of pregnancy-related symptoms. In addition, as the present case was pregnant with twins, compression symptoms were more likely to occur. Therefore, the diagnosis of gastric cancer during pregnancy was difficult in this case. However, if gastrointestinal symptoms are prolonged, or if symptoms such as severe weight loss, melena, a tender abdominal mass, or persistent and unexplained fever develop, an endoscopic assessment should be conducted to suspect other diseases.

Pregnancy-associated gastric cancer diagnosed after delivery is even more rare. Only three cases of gastric cancer diagnosed postpartum have been reported in the past 20 years [9-11]. Even with the three additional cases with postpartum diagnoses in Song et al.’s study [4], there are only six cases (Table 2).

**Table 2:** Cases of gastric cancer diagnosed postpartum in the past 20 years.

No.	Case	Age	Time of diagnosis	Symptoms	Stage	Survival outcome (time after diagnosis)
1.	Yildirim et al. [9]	29	2nd day	Abdominal pain, distention, fever, generalized peritonitis	Ⅳ	Death (6 months)
2.	Kurabayashi et al. [10]	31	5th day	Disseminated intravascular coagulation	Ⅳ	Death (13 days)
3.	Ebisui et al. [11]	28	5th month	Dysphagia	Ⅳ	Alive (13 years + 10 months)
4.	Song et al. [4]	26	28th day	Abdominal pain	Ⅳ	Death (2 months)
5.	Song et al. [4]	31	3rd day	Ascites	Ⅳ	Death (3 months)
6.	Song et al. [4]	31	10th day	Nausea, vomiting	Ⅳ	Death (23 months)

In Japan, postpartum women receive two routine checkups, though this is fewer than the 14 routine checkups conducted during pregnancy, making it even more difficult for obstetrician-gynecologists to recognize pregnancy-associated gastric cancer in the postpartum period. This patient presented with fever and upper respiratory symptoms during the influenza epidemic season and tested positive for influenza A. In consequence, the fever was initially considered to be due to influenza infection. However, when postpartum fever persists, as in this case, not only infections, such as influenza, intrauterine infection, and urinary tract infection but also malignant tumors must be considered for the differential diagnosis. Additionally, in this case, malignancy and metastasis should have been considered from an early stage due to the gradual elevation of lactate dehydrogenase, not just the effects of tissue damage caused by postpartum and cesarean section.

The five-year survival rate for pregnancy-associated cancer is low, and pregnancy-associated cancer has a high risk of death. By location, the adjusted hazard ratio for gastric cancer is higher than that for other types of pregnancy-associated cancer [12]. In 80% of cases, pregnancy-associated gastric cancer is already stage IV when it is diagnosed, with a reported curative resection rate of 27% and a three-year survival rate of 23.3% [3]. The patients have a poor prognosis, and early death after delivery has a significant impact not only on the patient but also on the child and family. Therefore, obstetrician-gynecologists should be attentive to symptoms associated with pregnancy during pregnancy checkups and need to suspect gastric cancer at an early stage to improve the prognosis.

Upper gastrointestinal endoscopy is generally used for screening gastric cancer and obtaining a tissue biopsy for definitive diagnosis, while the detection rate of gastric cancer under ultrasound scanning has also improved from 42.9% to 80.2% due to technological advances [13,14]. Gastric cancer during pregnancy is hardly visualized due to uterine enlargement but it was reported that diffuse wall thickening and luminal narrowing were able to be visualized even under abdominal ultrasonography during pregnancy [7]. In this case, abdominal ultrasonography was able to detect liver metastases, leading to the diagnosis of gastric cancer. Furthermore, abdominal ultrasonography is less invasive and easier to perform than endoscopy or CT and should be performed aggressively both during pregnancy and postpartum.

## Conclusions

Pregnancy-associated gastric cancer is rare and its prognosis is extremely poor. Gastric cancer symptoms during pregnancy are similar to those of hyperemesis gravidarum or pressure symptoms due to an enlarged uterus, making diagnosis difficult. If gastrointestinal symptoms are prolonged, or if symptoms such as severe weight loss, melena, a tender abdominal mass, or persistent and unexplained fever develop, an endoscopic assessment should be conducted to suspect other diseases such as gastric cancer. In addition, persistent fever should be considered not only infection but also malignancy, such as gastric cancer, which should be thoroughly investigated.

We performed conscientious abdominal examinations and abdominal ultrasound for the differential diagnosis of persistent postpartum fever and were able to identify multiple mass lesions in the liver, which led to the diagnosis of gastric cancer. Thus, intensive ultrasonographic assessment would help obstetrician-gynecologists to detect pregnancy-associated gastric cancer.

## References

[1] (2023). Cancer Information Service, National Cancer Center, Japan. Cancer statistics. (Article in Japanese). https://ganjoho.jp/reg_stat/statistics/stat/cancer/5_stomach.html.

[2] Sakamoto K, Kanda T, Ohashi M, Kurabayashi T, Serikawa T, Matsunaga M, Hatakeyama K (2009). Management of patients with pregnancy-associated gastric cancer in Japan: a mini-review. Int J Clin Oncol.

[3] Lee HJ, Lee IK, Kim JW, Lee KU, Choe KJ, Yang HK (2009). Clinical characteristics of gastric cancer associated with pregnancy. Dig Surg.

[4] Song MJ, Park YS, Song HJ (2016). Prognosis of pregnancy-associated gastric cancer: an age-, sex-, and stage-matched case-control study. Gut Liver.

[5] Katsuragi S, Tanaka H, Hasegawa J (2021). Analysis of preventability of malignancy-related maternal death from the nationwide registration system of maternal deaths in Japan. J Matern Fetal Neonatal Med.

[6] Tajima C, Ukita U, Matsuoka M, Kamihigashi M, Kato T, Harada K, Shibahara H (2020). Advanced gastric cancer diagnosed during the second trimester and treated with chemotherapy with S-1/L-OHP (SOX): a case report. (Article in Japanese). J Jpn Soc Perin Neon Med.

[7] Kubota H, Matsumoto H, Shimoya K (2011). Gastric carcinoma diagnosed due to vomiting and weight loss during pregnancy. (Article in Japanese). Stomach Intestine.

[8] Pacheco S, Norero E, Canales C, Martínez JM, Herrera ME, Muñoz C, Jarufe N (2016). The rare and challenging presentation of gastric cancer during pregnancy: a report of three cases. J Gastric Cancer.

[9] Yildirim Y, Erkan N, Avci E, Elveren B (2009). Perforated gastric cancer complicating early postpartum period of pregnancy. Acta Chir Belg.

[10] Kurabayashi T, Isii K, Suzuki M (2004). Advanced gastric cancer and a concomitant pregnancy associated with disseminated intravascular coagulation. Am J Perinatol.

[11] Ebisui C, Yamamura N, Minoji T (2019). Pregnancy-associated stage Ⅳ gastric cancer with no recurrence for a long time-a case report. (Article in Japanese). Gan To Kagaku Ryoho.

[12] Cairncross ZF, Shack L, Nelson G (2023). Long-term mortality in individuals diagnosed with cancer during pregnancy or postpartum. JAMA Oncol.

[13] Asai H, Oka H, Ogata K, Ichiyoshi M, Tanaka K (1981). Ultrasonographic diagnosis of gastric carcinoma. Japan J Gastroenterol Surg.

[14] Kitaura K, Kanawa T, Kansaku S, Honma Y, Yamamura K, Hirata N, Ito K (2014). Diagnostic ability of ultorasonography for gastric cancer—202 cases. (Article in Japanese). Japan J Med Ultrasound Technol.

